# Organochlorine pesticides, polybrominated diphenyl ethers and polychlorinated biphenyls in surficial sediments of the Awash River Basin, Ethiopia

**DOI:** 10.1371/journal.pone.0205026

**Published:** 2018-10-04

**Authors:** Niguse Bekele Dirbaba, Sen Li, Hongjuan Wu, Xue Yan, Jun Wang

**Affiliations:** 1 School of Environmental and Science and Engineering, Huazhong University of Science and Technology, Wuhan, China; 2 Key Laboratory of Aquatic Botany and Watershed Ecology, Wuhan Botanical Garden, Chinese Academy of Sciences, Wuhan, China; 3 Sino-Africa Joint Research Center, Chinese Academy of Sciences, Wuhan, China; National Sun Yat-sen University, TAIWAN

## Abstract

This study was initiated to document information on the levels of sediment contamination with organochlorine pesticides (OCPs), polychlorinated biphenyls (PCBs) and polybrominated diphenyl ethers (PBDEs). Moreover, it was intended to identify compounds which impose major ecological risks to aquatic organisms. Surficial sediments were collected from 46 locations within the streams and rivers of the Awash River Basin. In total 30 compounds were included in this study: 16 OCPs, 7 PCBs and 7 PBDEs. The total concentrations of OCPs, PCBs, and PBDEs ranged from 6.63 to 206.13 ng g^-1^- dry weight (dw), 0.85 to 26.56 ng g^-1^-dw and 3.71 to 18.95 ng g^-1^-dw respectively. Out of all the tested OCPs, heptachlor, heptachlor epoxide, p,p′-dichlorodiphenyltrichloroethane (p,p′-DDT) and β-hexachlorocyclohexane (β-HCH) were the most abundant in the study area. The ratio of (β-HCH/∑HCHs) indicated that HCHs were originally from earlier usage of HCH in the area whereas the ratio of (p,p’-DDT/p,p’-DDE) showed that the majority of DDT components were recently introduced into most of the sampling locations. Even though there were relatively low concentrations of PBDEs and PCBs across the sampling sites, substantial amounts of PCBs were observed in Addis Ababa City. According to the established ecological risk indices, p,p’-DDT and γ-HCH are the major concerns for potential adverse ecological impacts. This study provided the first comprehensive information on organohalogenated compounds’ (OCs’) occurrences, spatial distributions, and ecological risks in sediments of the Awash River Basin. Thus, the report will be very useful background information for further studies on sediment contamination with OCs’ in the region. It also adds important first-hand data to the field of fresh water ecology and provides useful empirical evidence for setting pollution control priorities for an ecologically important, yet largely understudied region.

## Introduction

Organohalogenated compounds (OCs) such as organochlorine pesticides (OCPs), polychlorinated biphenyls (PCBs) and polybrominated diphenyl ethers (PBDEs) were listed under persistent organic pollutants (POPs) in Stockholm Convention, 2001[1, 2]. These compounds were manufactured and used for different purposes (e.g. as pesticides, flame retardant additives, transformer dielectric fluids) before they were legally banned [3, 4]. Even though these products were produced for specific uses, they were also known for their adverse effects on the natural environment. It was clearly ratified that OCs are “poisonous to human beings, animals, plants and overall food webs” [5]. Exposure to OCs can cause hormone-dependent cancers, impair reproductive fitness and decrease the serum concentration of thyroid hormones in human beings and other animals [3, 6, 7]. Even at low concentrations, OCs can be a threat to natural ecosystems and human health as they are lipophilic, have low biodegradability, can easily accumulate in tissues and gradually cause chronic toxicities [8–10].

Although the use of most OCs has been banned due to their toxic nature, still they exist in the natural environments [5]. The majority of these compounds can be easily transported through the atmosphere and deposited at areas which are far away from their sources [11–12]. Moreover, OCs have a high affinity to soil and can easily get into water bodies through run-off from the surrounding terrestrial areas [13]. Agricultural runoff and urban flood storms are the secondary sources of streams and rivers contamination with POPs [14]. Due to these properties, the influences of POPs can be observed at local and regional levels. In water bodies, these compounds are easily adsorbed by sediments and suspended particulate matters because of their hydrophobic nature [15, 16]. Due to the sediment mobility, contaminants can be transported from rivers to other water ecosystem such as lakes and ocean and become threat to living organisms [17]. Thus, the river sediments can serve as a sink and potential source for OCs in aquatic environments [18].

Ethiopia has set a plan to become a middle-income country by 2025. Since agriculture is the leading economic sector in Ethiopia, much attention has been drawn to this sector by current growth and development policies. To promote agricultural productivity, the country has been importing massive quantity of pesticides and other agricultural inputs. Reports indicated that considerable amounts of pesticides are continuously imported to Ethiopia since the 1960s [19]. The data from 1996 to 2007 indicate that there has been an increasing trend in pesticide imports: 2973, 3670, 5079, and 8302 tons of pesticides were imported between the periods of 1996–1998, 1999–2001, 2002–2004 and 2005–2007 respectively [20, 21]. Moreover, high rates of urbanization and improper management of urban waste are contributing for the exposure to PCBs and PBDEs. Ironically, Ethiopia is a party for the Stockholm conventions to avoid or limit the production and use of persistent organic pollutants.

The Awash Basin is one of the most highly populated basins in Ethiopia with its rivers and streams receiving high volumes of untreated liquid and solid wastes from the capital city (Addis Ababa) and other large towns. About 15.7 million people were living in this basin by the year 2013 [22]. This accounts for 17% of the total population of the country. In the past two decades, expansion in agricultural fields, increases in exploitation, and competition on the water resources have been observed in this basin [23]. Such anthropogenic activities definitely cause water and sediment contamination with micro-pollutant such as OCPs, PCBs and PBDEs [24, 25]. Thus, it is crucial to establish a useful database and achieve a comprehensive understanding on the dynamics of the river’s ecosystem. For Awash Basin, although there are reports on contamination of agricultural soil [26, 27], there is no comprehensive data on freshwater sediment contamination with OCs.

This study was initiated to investigate the spatial distribution, concentration and compositional pattern of OCPs, PCBs and PBDEs in the sediment of streams and rivers of the Awash River Basin and to identify the possible sources of these pollutants. The study further evaluated the potential ecological risks of selected compounds to identify priority substances for future interventions. Generally the study will be useful to examine current conditions of the river and streams of the basin and prioritize possible future interventions.

## Materials and methods

### Description of the study area

**Fig 1:**
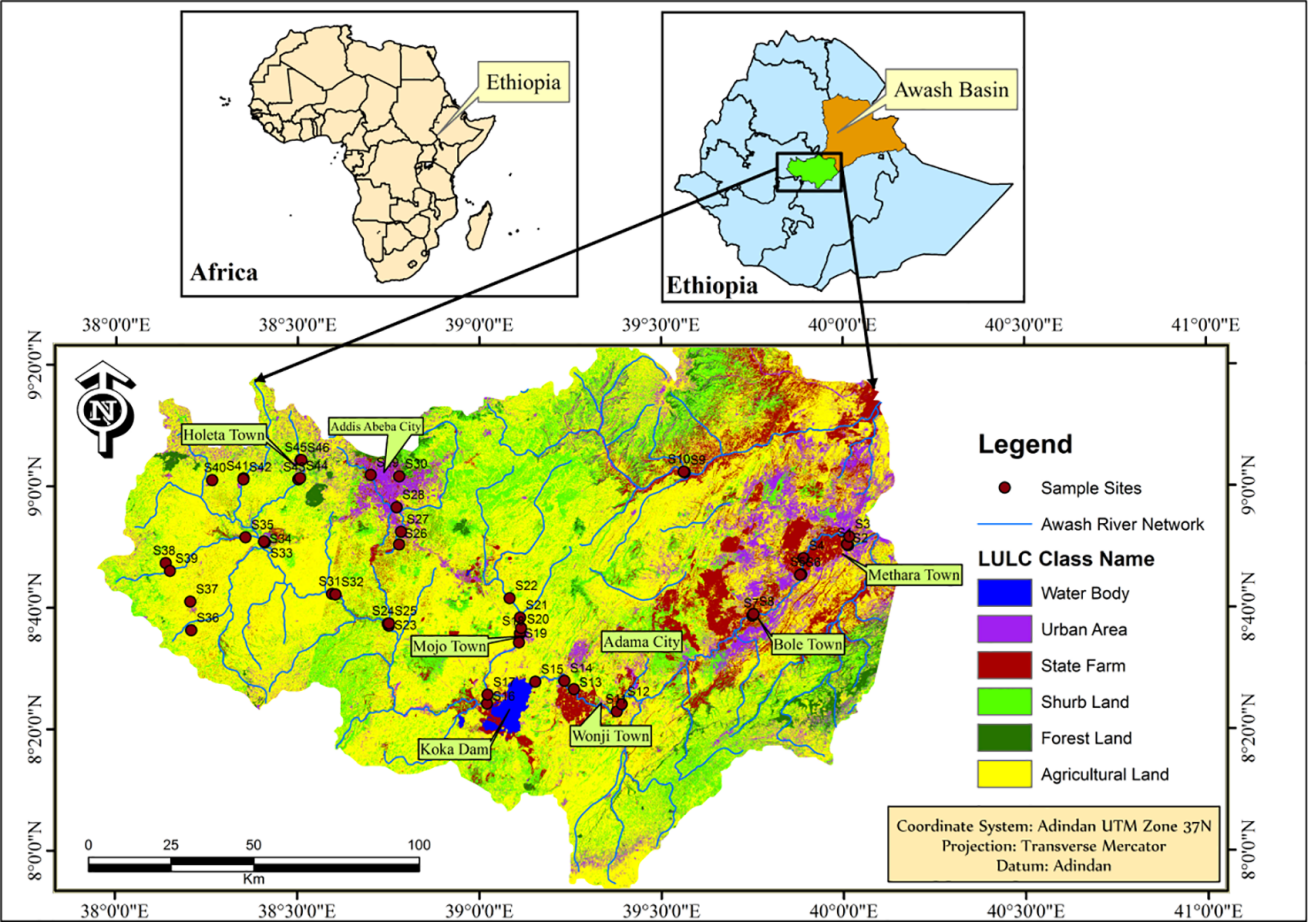
Map of the study area. Caption credit: Niguse BD, Yan X, Wu H, Colebrooke LL, Wang J (2018). Occurrences and Ecotoxicological Risk Assessment of Heavy Metals in Surface Sediments from Awash River Basin, Ethiopia. Water 10,535. https://doi.org/10.3390/w10050535.

The Awash River Basin is one of the 12 major basins of Ethiopia. The Awash River is 1200 km long and the catchment covers an area of 110,000 km^2^ [28]. The altitude ranges from 250 m in the east to 3000 m in the west. Even with such a wide altitudinal difference, the majority of the drainage areas in the basin are flat. The economic activities in the region are dominated by livestock production in the lower reach while mixed crop-livestock farming systems and urban livelihoods dominate in the middle and upper part of the basin. Sampling locations varied from the lower point (08^o^83’80”N, 040^o^01’38.6”E) on the main course of the Awash River up to the most upper point (09^o^07’308”N, 038^o^51’04.1”E) on the Kurane Stream within altitudinal range of 933.5 m to 2391 m. A map of the study area and locations of sampling sites are displayed in Fig 1 and S1 Table.

Before starting sample collection, preliminary field observation was conducted to determine about the specific sampling sites. Sampling sites were purposely identified in the manner which includes both area with limited human interference (e.g. Awash National Park and grazing lands) and sites with high level of modification (e.g. urban and industrialized zones). Accessibility and stream orders were also considered to determine the locations of specific sampling sites. Accordingly, representative sampling sites were located in urban, peri-urban and rural areas of the basin. Out of the existing river networks, the main course of Awash River, 4 major tributaries, 12 upper streams and Koka Dam were included in the sampling sites. No specific permissions were required for these sampling sites. The field studies did not involve endangered or protected species.

### Sample collection and preparation

The sediment samples were collected and prepared in August 2015; surficial sediment samples were collected from the top 0–10 cm using a metallic core sampler. Three grab samples (left side, right side and middle part of the rivers) were collected from each sampling site to make composite sample. The samples were packed with aluminum foil, transferred to an ice box and transported to a laboratory. All samples were air dried until constant weight. Gravels plant debris and other foreign materials were removed. The composite samples were sieved by using 2 mm metallic sieve and transported to China. The samples were further grinded by glass mortar; sieved using 0.3mm metallic sieve and stored at -4 ^o^C.

### Chemicals and materials

Copper powder, acetone, anhydrous sodium sulfate, florisil, dichloromethane and n-hexane (Fisher Scientific, USA) were used for extracting the compounds of interest. A mixture with known concentrations of 16 OCPs (4 hexachlorocyclohexane (HCHs) isomers: α-HCH, β-HCH, γ-HCH, δ-HCH; 3 dichlorodiphenyltrichloroethanes (DDTs): p,p'-DDE, p,p'-DDD, p,p'-DDT, heptachlor, heptachlor epoxide, α-endosulfan, β-endosulfan, endosulfan-sulfate, aldrin, endrin, endrin-aldehyde, methoxychlor), 7 indicators of PCBs (PCB-28, 52, 101, 118, 138, 153 and 180), 7 PBDEs (BDE-28, 47, 99, 100, 153, 154 and 183) and the internal standard pentachloronitrobenzene (PCNB) were purchased from AccuStandard (New Haven, CT, USA) to prepare laboratory standard solutions for machine calibration and quality control. To estimate the recovery rates, 2,2’,3,3’,4,4’,5,5’,6,6’-decachlorobiphenyl (PCB209) was purchased from Dr. Ehrenstorfer GmbH (Augsburg, Germany).

### Sample extraction

The sample extraction procedure was similar to the previous reports by [29, 30]. The sodium sulfate and silica gel were baked for 4 hrs at 450 and 180 ⁰C, respectively. Followed by the acidification with concentrated sulfuric acid (98.8%) at a 30% proportionality (wt/wt). Copper powder was rinsed by 2 mol of HCl for 12 hrs; repeatedly washed by distilled water and then by acetone to remove CuO. Washed Cu powder was stored under acetone until use. Cu was air dried to remove acetone and its powder was used to remove elemental sulfur during sample extraction. 1 g of ground sediment sample was thoroughly mixed with 3 g of C_18._ and ground for 5 minutes. Known volume of PCB-209 was spiked to each sample prior to grinding. Anhydrous sodium sulfate (1 g), Florisil (1 g), acidified silica gel (1 g), copper powder (1 g) and the ground mixtures were packed from bottom to top. Each column was eluted with known volume of dichloromethane (15 ml) in soxhlet apparatus. The extracts were concentrated, dried and re-dissolved in 100 μL n-hexane. Three replicates were extracted for each composite sample.

### Instrumental analysis and quality control

The design and implementation of instrumental analysis and quality control followed the method described in [30]. An Agilent 7890A gas chromatograph fitted with an electron capture detector (GC-ECD) and a Model 5975 mass spectrometer (MS) using electron-ionization ion source (EI) in the selected ion monitoring (SIM) mode (Agilent Technologies, Santa Clara, CA, USA) was used for this analysis. A capillary column HP-5MS (Agilent Technology, 30 m 0.25 mm i.d. 0.25 mm) was used to identify the compounds of interest. The flow rate of the carrier gas (helium) was attuned to 1.2 mL min^-1^. The three extracts of each composite sample were injected (1 μL) and the mean concentration calculated and used. The inlet was set at 280 ^o^C. The oven temperature was raised to 80 ^o^C within 1 min, gradually to 190 ^o^C (2 min) at 15 ^o^C min^-1^, then to 220 ^o^C (5 min) at 8 ^o^C min^-1^, and finally to 300 ^o^C (7 min) at 10 ^o^C min^-1^. The ion source and the detector were set to 300°C. The data were acquired and processed with Hewlett-Packard ChemStation software. The peaks of all compounds were identified by using the retention time of their respective standards (±1%).The detection limits were in the range of 0.001–0.3, 0.04–0.1, 0.02–0.05 ng·g^−1^ for OCPs, PCBs and PBDEs respectively. The percentage of total organic carbon (TOC) for each sediment sample was determined by using a solid TOC analyzer (Elementar, vario TOC cube, Germany). 5 mg of each sediment sample was used for TOC measurement.

A pair of matrix blank and sample spiked with 10 ng g^-1^ of all tested compounds was tested after every 20 samples to check the accuracy of the gas chromatograph. The average recovery rates for all tested compounds were within the range of 82 ± 11% to 104 ± 5%. The readings of solvent blanks were taken after 10 consecutive samples. PCB209 was spiked to all samples before extraction. Its average surrogate recovery in all samples was 76 ± 13%. The standard solutions with concentrations of 5, 10, 20, 50, 100, and 200 ng g^-1^of all tested compounds were used to make the standardization curves. The relative correlation coefficient (r) of standardization curve for all compounds were greater than 0.993.

### Guidelines for ecological risk assessment

Two sets of sediment quality guidelines (SQGs), the effects range-low and effect range-median (ERL–ERM) and threshold effect level and probable effect levels (TEL–PEL), were used for ecological risk assessment of sediment of the Awash River Basin. The first guideline (ERL–ERM) was developed by [31] while the second approach (TEL–PEL) was established by [32]. ERL and TEL values were proposed to represent concentrations below which adverse impacts were not frequently expected, and above which effects may begin [9]. Whereas ERM and PEL values describe the concentrations above which adverse effects are more likely to occur [33]. Values between ERL–ERM and TEL—PEL indicate potential adverse effects [31, 32].

### Statistical analysis

The statistical analysis was conducted by statistical package for social sciences (SPSS) version 20. The relationships between the tested OCPs (ng g^-1^-dw) and TOC (%) were defined by using a Pearson correlation analysis. A *p* < 0.05 was considered statistically significant. In addition to correlation analysis, principal component analysis (PCA) was implemented to identify potential sources of contaminants. Varimax rotation method was used to rotate the component matrixes. Graphs which indicate the mean distributions of the tested compounds and percentage proportion of selected compounds were produced by using Origin Pro8 1997–2007.

## Results and discussion

### Distribution and compositional profiles of OCs

#### Concentrations of OCPs, PBDEs and PCBs in the river sediments

The concentrations of determined OCPs are summarized in Table 1. The mean value for 16∑OCPs at the study area was 76.43 ng g^-1^- dw (range 6.63–206.13 ng g^-1^- dw). Out of all tested OCPs, the three highest mean values (24.19, 23.77 and 10.6 ng g^-1^- dw) were recorded for heptachlor (range: n.d. - 42.88 ng g^-1^- dw), heptachlor epoxide (range: 0.52–36.29 ng g^-1^- dw) and p,p'-DDT (range:1.60–108.87 ng g^-1^- dw) respectively. High hydrophobicity of the heptachlor can increase its chance of accumulation in the surficial sediments [34]. Even though there are variations among sampling sites, this property might have contributed for high concentration of heptachlor in the study area. Moreover, most of the samples were collected from rift valley area where termites are the major problems. Thus, intensive use of heptachlors to control the termites might have also contributed for high accumulation of this compound in the river sediments. High proportion of p,p'-DDT can occur in the sediments due to its low biodegradability [35].

**Table 1:** Descriptive statistics for the concentrations of OCPs in the sediments of rivers and streams of the Awash River Basin (ng g^-1^-dw) (N = 46).

Chemicals	Range	Mean	Median	RSD (%)	Det. Freq.
α-HCH	0.86–3.10	1.52	1.36	30.66	100%
β-HCH	0.71–12.24	5.68	5.65	52.60	100%
γ-HCH	n.d. -1.54	0.91	1.03	43.99	93.5%
δ-HCH	n.d. - 1.14	0.22	0.16	119.09	73.91%
∑ HCHs	2.41–15.90	8.33	7.98	41.63	100%
p,p'-DDE	n.d. - 4.46	1.11	0.87	8210	91.3%
p,p'-DDD	n.d. - 29.31	2.27	1.49	186.07	97.8%
p,p'-DDT	1.60–108.87	10.60	5.19	159.68	100%
∑DDTs	1.99–139.68	13.98	7.54	152.59	100%
heptachlor	n.d.– 42.88	24.19	24.97	33.33	97.8%
heptachlor epoxide	0.52–36.29	23.77	25.08	40.92	100%
α-endosulfan	n.d. - 1.34	0.46	0.55	67.53	80.4%
β-endosulfan	n.d. - 0.9	0.35	0.60	90.68	34.8%
∑endosulfans	n.d– 2.08	0.81	0.74	61.19	89.1%
endosulfan-sulfate	n.d. - 7.21	1.85	1.83	61.76	89.1%
aldrin	n.d. - 2.31	0.57	0.57	81.14	73.9%
endrin	n.d. - 1.91	1.00	1.34	68.46	73.9%
endrin-aldehyde	n.d. - 1.45	0.78	0.78	65.34	80.43%
methoxychlor	n.d. - 2.94	1.41	1.26	68.23	76.08%
∑ Others	1.33–10.26	5.34	5.28	32.00	100%
∑OCPs	6.63–206.13	76.43	73.66	40.54	100%

Where ∑ Others = endosulfan-sulfate + aldrin + endrin + endrin-aldehyde + methoxychlor ∑OCPs = the concentration for 16 tested organochlorine compounds.

Among, HCH isomers, highest mean value (5.68 ng g^-1^- dw) was recorded for β-HCH (range: 0.71–12.24). This indicates that other isomers were degraded to β-HCH. Though the HCH had been used widely as a pesticide in agricultural sector in most of the developing nations [36], the sum of HCHs was found to be less abundant than heptachlor, heptachlor epoxide and the sum of DDTs in this study area. HCHs have higher biodegradability and vapor pressure that can lower its accumulation in the sediments” [37, 2]. The same property might have affected the distribution of HCHs in the Awash River Basin. The detection frequencies for some OCPs were as high as 100% indicating their wide incidence.

The concentrations of PCBs and PBDEs compounds are summarized in Table 2. The concentration of PBDEs (∑_7_PBDEs) reclined in the ranged of 3.71–18.95 ng g^-1^-dw with an overall mean value of 7.33 ng g^-1^-dw. Out of the tested PBDE congeners, BDE-47 showed a relatively wide range of concentrations (0.61–12.51 ng g^-1^-dw). The sum of PCB compounds (∑_7_PCBs) ranged from 0.85–26.56 ng g^-1^-dw with a mean of 10.20 ng g^-1^-dw and median of 9.62 ng g^-1^-dw. Thus, low concentrations of ∑_7_PBDEs and ∑_7_PCBs were observed in the study area. This might show a relatively low consumption of these products. Similar concentrations of PCB were reported from Danube Delta and Nile River sediments [38, 39].

**Table 2:** Concentrations (ng g^-1^-dw) of PBDEs and PCBs in Awash River Basin (N = 46).

Chemicals	Range	Mean	Median	RSD (%)	Det. Freq.
BDE-28	n.d-2.22	0.96	1.00	57.76	80.43
BDE-47	0.61–12.51	1.35	0.67	171.13	100.00
BDE-99	0.611–5.913	1.01	0.70	83.18	100.00
BDE-100	n.d.-1.22	0.68	0.67	20.28	100.00
BDE-153	0.65–1.21	0.76	0.74	12.79	100.00
BDE-154	n.d-1.44	0.85	1.00	47.30	84.78
BDE-183	n.d.-4.96	1.72	1.74	70.37	80.43
∑PBDEs	3.71–18.95	7.33	6.99	40.17	100.00
PCB-28	n.d—4.84	1.62	1.71	72.57	82.61
PCB-52	n.d -18.91	3.69	2.33	104.35	91.30
PCB-101	n.d—3.75	1.67	1.78	58.95	89.13
PCB-118	n.d—1.55	0.47	0.50	46.84	93.48
PCB-138	n.d—2.04	0.37	0.35	97.00	78.26
PCB-153	n.d—4.80	0.57	0.45	127.05	80.43
PCB-180	n.d—12.45	1.82	1.32	101.73	93.48
∑PCBs	0.85–26.56	10.20	9.62	49.70	100.00

Even though the concentrations were found to be low at most sampling sites, most of the PBDEs congeners were detected across all sampling sites (detection frequency = 100%). Transportation through aerosol and gradual deposition of the aerosols could have contributed for a wide distribution of PBDEs compounds [40].

#### Comparison of selected OCs concentration with previous studies

The use of OCs has been banned or controlled across the world, but reports indicate high accumulation of these compounds in different parts of the natural environment [41, 42]. A comparison between this study and other similar reports on OCs concentrations is presented in Table 3. HCH’s concentration in the Awash River basin was similar to its concentrations in the Soan River, Pakistan [43]. A wide range of concentrations of HCH was recorded in this study as compared to report on the surface sediments of the Rivers in middle Denube, Serbia [44] and the Yellow River, China [45]. In contrast, the range of HCH concentration in this study area was found to be lower than a report from the Qiantang River, China [15].

**Table 3:** The comparison of OCs concentrations (ng g^-1^-dw) in surficial sediment with results from different parts of the world. The values between parentheses are mean values.

Compounds	Locations	Year	Concentration	Reference
4HCHs	Soan River, Pakistan	2008–9	5.79–17.2	[43]
4HCHs	Rivers in middle Denube, Serbia	2014	n.d-1.03	[44]
4HCHs	Yellow river, China	2008	0.05–5.03	[45]
4HCHs	Qiantang River, China	2006	19.74–152.1 (44.1)	[15]
4HCHs	Awash River Basin, Ethiopia	2015	2.41–15.9 (8.33)	The study
3DDTs	Qiantang River(China)	2006	8.64–100.2 (25.13)	[15]
3DDT s	Rivers in middle Denube (Serbia)	2014	0.455–61.2	[44]
3DDTs	Soan River, Pakistan	2008–9	6.98–30.1	[43]
3DDTs	Huaihe River, China,	-	n.d.– 10.37(2.43)	[46]
3DDTs	Awash River Basin, Ethiopia	2015	1.99–139.68	The study
11OCPs	Rivers in middle Denube, Serbia	2014	0.564–61.6	[44]
25OCPs	Lake Qarun, Egypt	2011	1.01–164.8	[47]
13OCPs	Soan River, Pakistan	2008–9	47.39	[43]
16OCPs	Awash River Basin, Ethiopia	2015	6.63–206.13 (76.43)	The study
9PBDEs	Zhujiang River, China	-	1.1–49.3(12.9)	[48]
7PBDEs	Cinca River, Spain	2002	2–41.7	[49]
9PBDEs	Niagara River, North America	2003	0.72–148.0	[50]
7PBDEs	Awash Basin, Ethiopia	2015	3.71–18.95(7.33)	This study
11PCBs	River Nile (Egypt)	2013	1.68–2.46	[39]
7PCBs	Huveaune River France	2010	2.8–435	[51]
8PCBs	Umgeni River, South Africa	2013	102.60–427.8	[52]
7PCBs	Awash Basin, Ethiopia	2015	0.85–26.56 (10.2)	This study

The concentration of ∑DDTs was lower than reports in the Qiantang River, China [15], the Rivers in middle Denube, Serbia [44] and on the Soan River, Pakistan [43]. Whereas higher concentration of ∑DDT was recorded in this study area as compared to reports on the Huaihe River, China [46]. The total sum of OCPs showed a higher range than values in Lake Qarun, Egypt [47], the Rivers in middle Denube, of Serbia [44] and in Soan River, Pakistan [43]. The sediments from Awash River Basin were found to be less contaminated with PBDEs compared with reports from Zhujiang River of China [48], Cinca River of Spain [49] and Niagara River, North America [50]. The PCBs concentration of the study area was higher than reports from River Nile in Egypt [39]. Lower concentration of PCBs was detected in the Awash River Basin compared with the Huveaune River of France [51] and the Umgeni River of South Africa [52].

#### Spatial distribution of OCPs, PBDEs and PBCs in the river sediments

**Fig 2:**
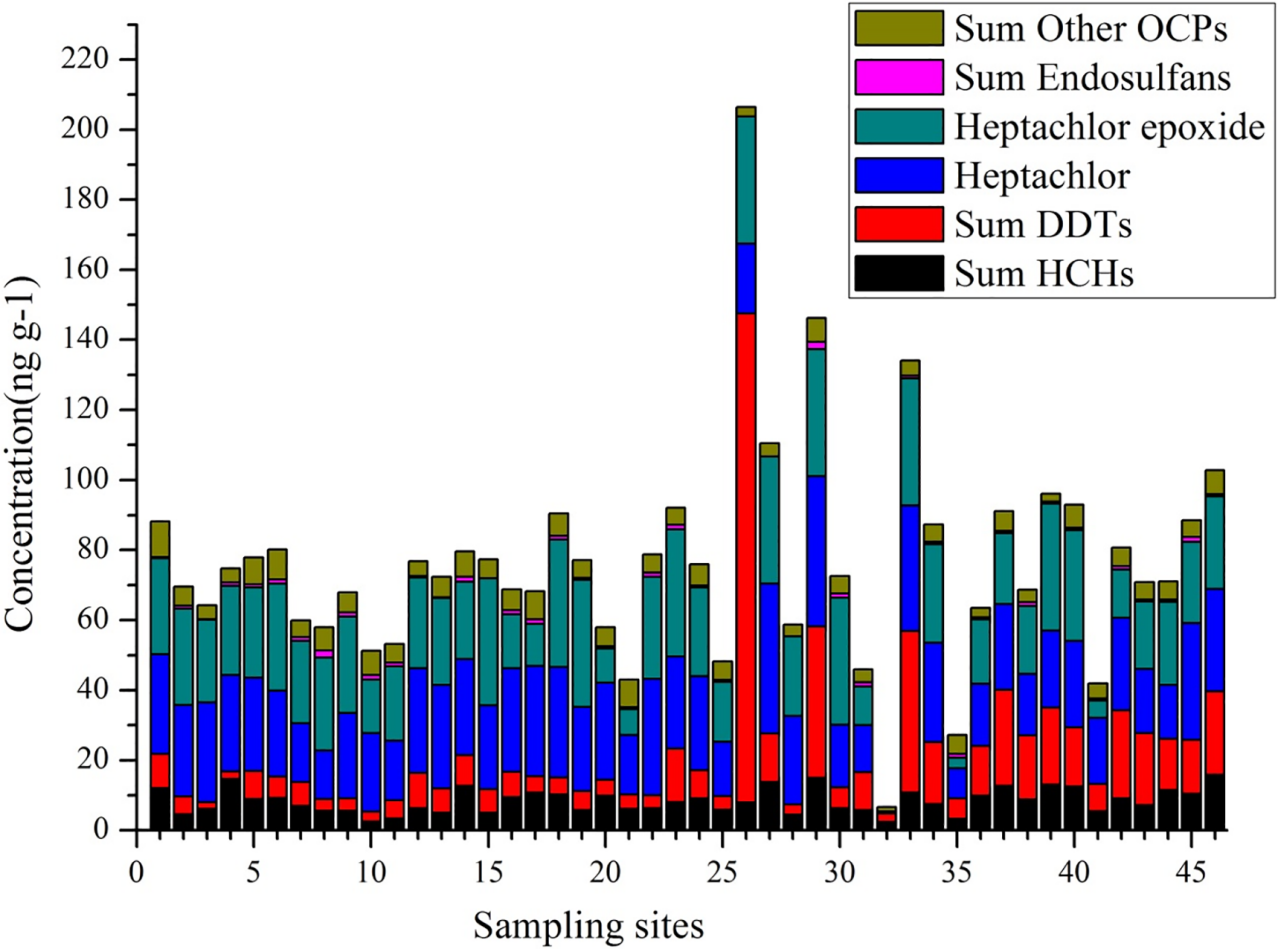
The concentration (ng g^-1^ dw) of 16∑OCPs at each sampling site across the study area. Where ∑Others = endosulfan-sulfate + aldrin + endrin + endrin-aldehyde + methoxychlor.

The concentrations of OCPs in the sediments of the Awash River Basin were displayed in Fig 2 and S2 Table. The highest total OCPs concentration was observed at site 26 (206.13 ng g^-1^- dw), followed by site 29 (146.20 ng g^-1^- dw), and site 34 (134.09 ng g^-1^- dw). The first two sites by the Akaki River were located at a peripheral zone of the capital city (Addis Ababa) where vegetable farms and solid waste dump were nearby. The third site was located in the Awash River at a point where large extents of farmlands and villages are frequently affected by floods. Similarly, the highest three values of ∑DDTs were also recorded at these three sites: site 26 (134.09 ng g^-1^- dw), site 33 (46.15 ng g^-1^- dw) and site 29 (43.2 ng g^-1^- dw). Thus, runoffs from the peri-urban areas and waste discharge from the capital city could be the major sources of these organic compounds. For site 33, runoff from the nearby floriculture farms could be the main cause for high accumulation of OCPs.

The three highest ∑HCHs concentrations (15.9, 14.96, and 13.55 ng g^-1^- dw) were recorded at sites 46, 29 and 27 respectively. The first site was located in the Kurane River and close to a demolished floriculture farm. Surface runoff from this farm could be a major source of old HCHs. Some OCP congeners (i.e aldrin, endrin, endrin-aldehyde and methoxychlor) showed relatively similar concentrations across the sampling sites. This may indicate that diffused non-point sources such as runoff from agricultural fields, villages, and urban areas could be their major sources.

**Fig 3:**
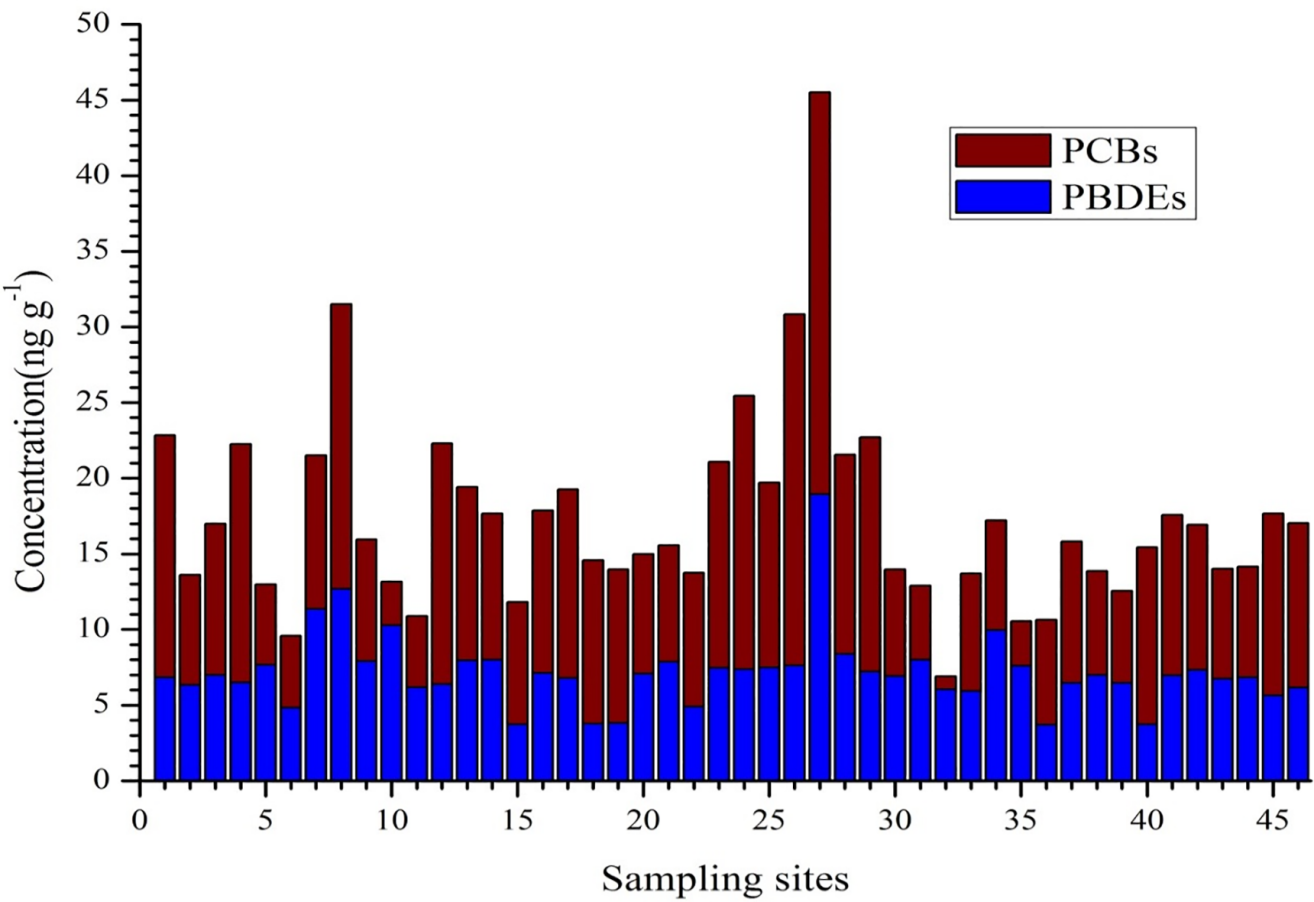
The concentration (ng g^-1^ dw) of 7PBDEs and 7PCB across sampling sites.

The highest PBDEs concentration was recorded at site 27 (on Akaki River in Addis Ababa City) followed by two consecutive sites along the Awash River (site 8 and 7) in Bole Town (Fig 3 and S2 Table). The pronounced contaminations were observed only in the sediments that were collected from an urban area. This might be related to the waste discharge from factories such as textile and foam factories which were using PBDEs as additives [53] and uncontrolled electronic waste disposal. Gentle slope and extensive sedimentation in the Bole area might have also contributed for gradual accumulation of these contaminants in the case of site 8 and 7.

**Fig 4:**
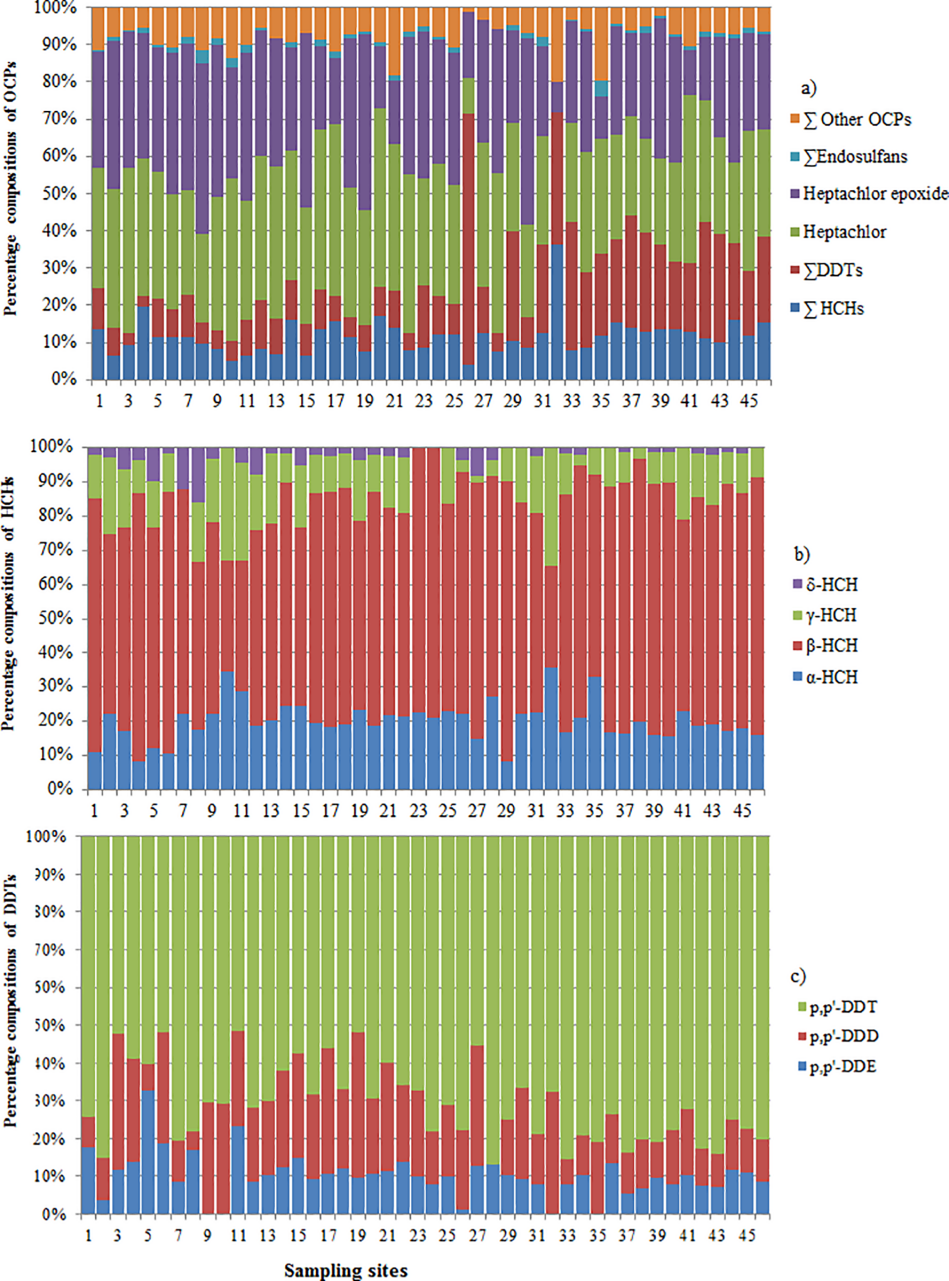
Compositional patterns (%age) of OCPs in the sediments of Awash River Basin. Where a) = the overall % proportion for test 16 OCPs; ∑other OCPs = the sum of % contributions of endosulfan-sulfate + aldrin + endrin + endrin-aldehyde + methoxychlor; ∑Endosulfans = the sum of % contributions of α- and β- endosulfan b) = hexachlorocyclohexane isomers (HCHs), c) = DDT analogues.

Alike PBDEs, the highest PCBs concentration (26.56 ng g^-1^-dw) was also found at site 27. PCB concentration below 22.7 ng g^-1^-dw not frequently cause adverse ecological impacts [31, 54]. This study showed that only samples from 2 sites (sites 26 and 27) had exceeded 22.7 ng g^-1^-dw (Fig 4 and S3 Table). This indicates low surface sediment pollutionwith 7PCB congeners and also suggests low eco-toxicological concerns from these compounds.

However relatively low concentrations of PBDE and PCB compounds were observed in the basin, samples collected from Akaki River (site 26 and 27) showed signals of ecological risk from sediment pollution with PCB. About 4,300 tons of e-waste was confined in 10 major cities of Ethiopia and mainly in Addis Ababa [55]. Therefore, this result can be used as an early warning for sediment contamination with PBDE and PCB. The distribution of both PBDE and PCB did not show any regular trend across the sampling sites. This might show non-homogeneous inputs of these chemicals and/or influence by variations in hydrological features of the rivers [54].

#### Compositional patterns of OCPs, PBDEs and PCBs

The differences in compositional patterns of HCHs isomers or DDTs congeners in the natural environment could indicate variability in application history, sources of contamination, and transport mechanisms [16, 56]. The results indicate that heptachlor and heptachlor epoxide cover the highest portions of 16 tested OCPs at most sampling sites (Fig 4). The HCHs isomers and DDTs had considerable proportions while other OCPs compounds had accounted for relatively a minor proportion. In this study the highest % proportion of HCHs was contributed by β-HCH (68.18%) followed by α-HCH (18.26%), and γ-HCH (10.97%). β-HCH is known for its high persistence and poor biodegradability in soil and sediment [57–59] and these properties might have contributed for its abundance in the river sediments of the Awash River Basin. [58] reported that α-HCH and γ-HCH can be biodegraded to β-HCH in the natural environment. Thus conversion of α-HCH and γ-HCH to β-HCH through microbial degradation might have contributed for high concentrations of β-HCH in this study area. The ratio of (β-HCH/∑HCHs) at these sampling sites was ranged between 0.33–0.82. About 93.47% of the sites had β-HCH/∑HCHs ratios above 0.5. This indicates that β-HCH is the dominant HCH congener in most sediment samples. Moreover, β-HCH and α-HCH contributed about 88.12% of the total HCHs whereas α-HCH contributes only 18.26% indicating that the majority of HCHs were originally from old deposits of HCHs.

The lower percentage of α-HCH in this study indicates a low chance of recent application of technical HCH in most sites of the study area. Since α-HCH is biodegradable to β-HCH under aerobic condition [56], a high percentage of α-HCH could indicate a recent use of technical HCH and vise-versa. For technical grade HCHs, the ratio of α-HCH/γ-HCH varies between 3 and 7, whereas values close to zero indicate recent application of γ-HCH (lindane) [60, 61]. In this study the ratios of α-HCH/γ-HCH only 15.22% of the sampling sites showed the ratio between 3 and 7 while the remaining sites sowed ratio far below 3. This indicates the application of both technical mixtures and lindane even though the majority of HCHs originated from lindane. The high observation of low α-HCH/γ-HCH ratio might be associated with the fact that lindane was banned recently in 2011.

The ratio of p,p’-DDT/p,p’-DDE ranged from 2.21 to 11.7 for all sampling sites except for site 26 (with maximum DDT concentration) which was 73.11. The ratio of p,p’-DDT/p,p’-DDE can be used as an index to identify the use of fresh and/or aged DDT at a given site [62]. A value < 0.33 generally indicates an old deposit [63]. Thus, this study indicates a recent application of DDTs in all sampling sites. This most likely happened due to a recent use of DDTs for controlling vector borne diseases and mosquitoes in the region [64]. Surface runoff during sampling period might have also contributed to a contamination with fresh DDTs. The ratios of (DDE +DDD)/DDTs at most sampling site were < 0.5. Previous studies by [65, 66] stated that (DDE +DDD)/DDTs < 0.5 indicate recent application of DDTs. The ration of (DDE +DDD)/DDTs > 0.5 indicates a long weathering of DDTs [56].

**Fig 5:**
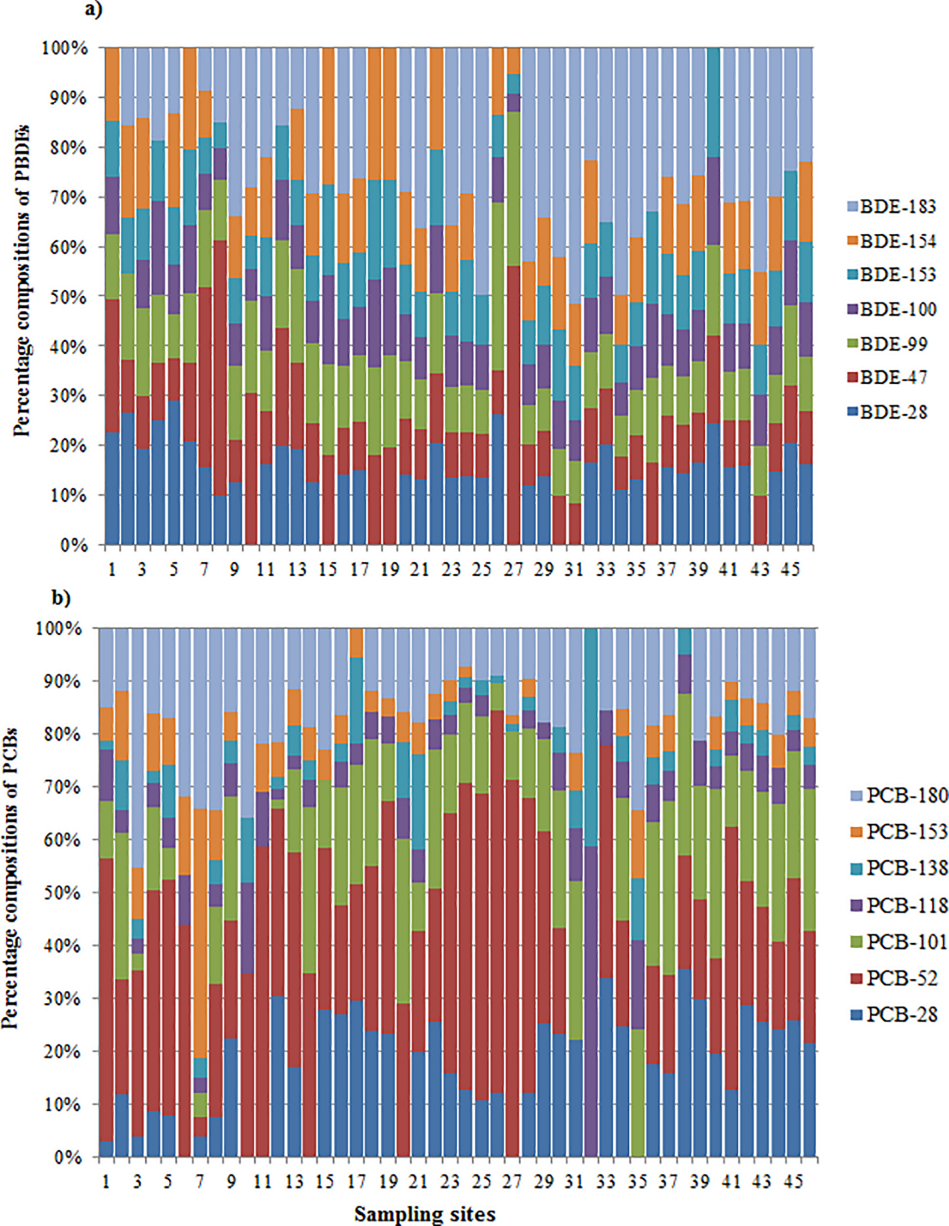
Compositional patterns (%age) of PBDEs and PCBs congeners in the sediments of Awash River Basin.

Fig 5 indicated that BDE-183 contributed the highest median concentration (24.8%) of the total PBDEs followed by BDE-28 (14.4%), BDE-154 (14.3%), BDE-153 (10.6%), BDE99 (10.1%), BDE47 (9.6%) and BDE100 (9.6%). The high proportional contribution of BDE183 indicates utilization of octa-BDE products [67]. Therefore, occurrence of a comparatively dominant proportion of BDE-183 indicates the likelihood of anthropogenic inputs of octa-BDE products [68]. Presence of BDE-183 indicates the use of PBDEs’ mixtures in the nearby industrial areas [69]. Complex patterns of PBDEs congeners were observed across the study area. This might be associated with variation in sources, dispersion, accumulation patterns and metabolism by benthic communities [70].

The PCBs were not uniformly distributed across the sampling sites. PCB-52 (tetra-PCB) contributed the maximum proportions of PCBs (72.34% and 71.21%) at sites with the highest concentrations of PCBs (site 26 and 27 respectively). Similarly, the sum of two compounds tri and tetra PCBs (PCB-28 and PCB-52) contributed (50.8%– 84.6%) of the total PCBs concentrations at about 50% of the total sampling sites. A similar report on river sediments in Hong Kong shows high enrichment with low chlorinated PCBs, (sum of mono, tri- and tetrachlorobiphenyl) contributed more than half of total PCBs [71]. Low contributions of high chlorinated PCBs such as PCB-180, 153,138 and 118 might indicate that these compounds probably settled onto the bottom sediment immediately near to the source [63].

### The relationships between OCs and TOC

The results obtained from the TOC analyses indicated that almost all sediment samples had relatively low percentages of TOC. The correlation analysis (Table 4) indicated that there were weak correlations between TOC and tested OCs. This might be attributed to human activities that could have resulted in disequilibrium [66, 72]. Among all the tested OCs, only ∑ DDTs (r = 0.436**, P = 0.01) and ∑HCHs (r = 0.305*, p = 0.05) showed significant positive correlation with TOC. This might indicate that most of the tested OCs were less adsorbed by organic carbon components and might have different transportation mechanisms. Similarly a weak correlation between TOC and OCs was reported by [68] in Beijiang River, China. Report by [49] pointed out that such a weak correlation could be associated with the mixing mechanisms and the nature of uncontaminated sediments. Moreover, continuous input of fresh OCs could also affect its correlation with TOC [68]. The significant positive correlations between ∑HCHs and heptachlor (r = 0.560**, P = 0.01), heptachlor and heptachlor epoxide (r = .536**, P = 0.01), heptachlor epoxide and ∑DDTs (r = .308*, p = 0.05) indicate similarities in their distribution patterns and/or presence of their common potential sources. Application of agricultural pesticides could be the possible way for their entrances to the natural environment. Soil erosion due to heavy rainfall is one of the major secondary sources of sources of the POPs especially in tropical areas [73]. Since the sampling time of this study was in the wet season, runoff from agricultural lands could be major source of these OCPs. Relatively weak correlation between ∑DDTs and other OCs indicates that ∑DDTs had other additional source. In Africa DDT was used for controlling mosquitoes [74]. Utilization of DDTs in the health sectors to control vector borne diseases such as malaria could be its major source. Sum endosulfuns showed weak correlation with other OCs. Endosulfuns had been used as acaricide in livestock production sector [75, 76]. Thus, waste from livestock farms could be one of its major sources. Insignificant correlations were observed between PBDE and OCPs congeners. This may also point out that these compounds have different origins. The ∑PBDEs showed significant positive correlation (r = 0.433**, p = 0.01) with ∑PCBs and some PCBs congeners indicating possible common anthropogenic sources and/or the transport path way of these groups of chemicals. Open burning of waste at dumping sites and effluents from industrial areas are among the potential sources of both PBDEs and PCBs [77]. Moreover, PCBs showed significant positive correlation with heptachlor (r = 0.488**, P = 0.01). Even though there is similar report in soil samples of Kenya [45], the basic reason for this strong positive correlation between OCPs and of PCBs was not clearly justified.

**Table 4:** Pearson correlations between OCs concentrations and TOC (%).

	∑HCHs	∑DDTs	helor	heoxide	∑ensul	∑others	PBDEs	PCBs	TOC
∑HCHs	1								
∑DDTs	0.238	1							
helor	0.560**	0.100	1						
heoxide	0.358*	0.308*	0.536**	1					
∑ensul	0.115	-0.153	0.091	0.019	1				
∑others	0.205	-0.243	0.293*	0.041	0.365*	1			
PBDEs	-0.004	-0.005	0.105	-0.028	0.015	-0.033	1		
PCBs	0.391**	0.375*	0.488**	0.418**	-0.133	0.029	0.433**	1	
TOC	0.305*	0.436**	0.034	0.064	-0.054	-0.328*	0.089	-0.009	1

** = Correlation is significant at the 0.01 level (2-tailed)

* = Correlation is significant at the 0.05 level (2-tailed), helor = heptachlor, heoxide = heptachlor epoxide, ∑ensul = sum of endosulfuns.

Principal component analysis (PCAs) was performed for the groups of OCs and TOC (Table 5). Four principal components with Eigen values greater than one were used to describe the significance of the principal components (PCs). PCAs with Eigen values greater than 1 constituted for 75.01% of the total variations. Loading values > 0.7 was used to represent the strong correlation between components and the original variables.

**Table 5:** Result of PCA for the OCs and TOC in the surface sediment from the Awash River Basin.

Variables	PC1	PC2	PC3	PC4
∑HCHs	**0.716**	0.357	0.326	-0.003
∑DDTs	0.353	0.639	-0.316	0.010
heptachlor	**0.828**	-0.046	0.202	0.116
heptachlor poxide	**0.791**	0.060	-0.121	-0.098
∑endosulfans	-0.029	0.023	**0.850**	0.003
∑others	0.279	-0.454	0.637	-0.039
PBDEs	-0.027	0.040	0.041	**0.964**
PCBs	0.670	0.017	-0.218	0.578
TOC(%)	-0.006	**0.923**	0.046	0.038
**Eigen values**	**2.421**	**1.602**	**1.441**	**1.29**
**% of total variance**	**26.9**	**17.8**	**16.01**	**14.34**
**% of cumulative total variance**	**26.9**	**44.7**	**60.71**	**75.04**

The disparity in dominance categories during principal component analysis may specify the difference in source of pollutants. PC_3_ showed the dominance of sum endosulfans. This also indicates that endosulfans has unique source of sediment contamination with endosulfans. The correlation analysis also indicated that endosulfan have weak correlation with other OCs. PC4 was positively correlated with high loading value of PBDEs. This also indicates that PBDEs has no similar sources with all OCP compounds. Electronic wastes such as televisions, computers and mobiles are their possible sources of PBDEs [51].TOC, ∑DDTs, PCBs and ∑other OCPs showed loading values <0.7 in all PC indicating that these compounds have different major sources for sediment pollution.

PC1 describes 26.9% of the total variations. Heptachlor, heptachlor epoxide and ∑HCHs were dominant (loading value > 0.7) in PC_1_. The occurrence of such compounds in the same PCs could indicate the similarity in their origin [9]. These groups of OCs were mainly used as pesticides in agricultural sector. PC_2_ contributing for 17.8% of the total variances and influenced by high loading of the TOC. The correlation analysis also showed weak correlation between TOC and OCs which infers to low impact of TOC on OCs distribution.

### Ecological risk assessment for selected OCPs and PCBs

The concentrations (ng g^-1^-dw) which pose ecological risk to aquatic organisms were summarized in Table 6 based on the effects range-low and effects range-median (ERL–ERM); threshold effect level and probable effect level (TEL–PEL) sediment quality guidelines. These descriptions rely upon analyses of matching chemical and biological effects data compiled from previous studies [31, 32, 78, 79]. The mean concentrations of p,p’–DDT and ∑DDTs were above their respective ERL values at all sampling sites whereas p,p’-DDE, p,p’–DDD and γ-HCH showed mean concentration above their respective ERL at about 10.87, 28.26, and 80.43% of the sampling sites respectively. The concentrations of DDTs compounds were lower than ERM values in most sampling sites whereas γ-HCH was higher than its respective ERM value at most sampling sites (63.04%). The concentration of p,p’ -DDT and γ-HCH were > PEL value at most sampling sites (>56.76%) whereas p,p’–DDD and p,p’-DDE had concentrations less than PEL at almost all sampling sites (>97.%). Thus, adverse ecological effects are expected to frequently occur mainly due to p,p’–DDT and γ-HCH.

**Table 6:** Evaluation of potential ecological risk of DDTs, γ-HCH and ∑PCBs in surface sediments of the Awash River Basin using TEL, PEL, ERL and ERM guideline values (ng g^-1^-dw).

Compounds	Concentration	ERL	>ERL (%)^a^*	ERM	>ERM (%)^a^*	TEL	>TEL (%)^a^*	PEL	>PEL (%)^a^*
p,p’-DDE	n.d. - 4.46	2^a^	10.87	15^a^	0	2.1^b^	10.87	374.2^b^	0.00
p,p’ -DDD	n-29.31	2^a^	28.26	20^a^	2.17	1.2^b^	63.04	7.8 ^b^	2.17
p,p’ -DDT	1.60–108.87	1^a^	100	7^a^	41.3	1.2^b^	97.83	4.8 ^b^	56.52
∑DDTs	1.99–139.68	1.6^b^	100	46 ^b^	4.35	3.9^b^	82.61	51.7 ^b^	2.17
γ-HCH	n-1.54	0.32^d^	80.43	1^d^	63.04	0.32^d^	80.43	0.99^d^	63.04
∑PCBs	0.85–26.58	22.7 ^c^	4.35	180 ^c^	0	-		-	

Where the superscripts ^(a b c d)^ indicate reference materials

^a =^ [78]

^b =^ [32]

^c =^ [31]

^d =^ [79]

ERL = effects range-low value; ERM = effect range-median value; TEL = threshold effect level value; PEL = probable effect level value

^a^* = Percent samples above corresponding limits.

The ∑PCBs concentrations above ERL value were observed only at two sampling sites (sites 26 and 27). These two sites were located in the Akaki River in and around the capital city.

The ∑PCBs concentrations at all sampling sites were considerably lower that ERM value. Thus, within the current condition, adverse impacts are rarely expected to occur due to sediment contamination with PCBs and the toxicity of PCBs in sediments of the study can be classified as low. PBDEs are not included in ecological risk assessments due to lack of their background values for ERL, ERM, TEL and PEL.

## Conclusions

This study provides the first comprehensive information on occurrence, spatial distribution and ecological risk of POPs in sediments of the Awash River Basin. Even though low concentrations of PBDEs and PCBs were detected in the sediments, the prevalence of high concentrations of OCPs indicates that sediment pollution with POPs is a major ecological issue in the Awash River Basin. Among OCPs, heptachlor was the most abundant contaminant. This points out that heptachlor was widely used as an insecticide. β-HCH and p,p’-DDT were the dominant components of HCHs and DDTs, respectively. Therefore, it is likely that, HCHs were sustained in the sediments from aged deposit while the technical DDT was recently introduced. The distributions of all tested OCs were weakly influenced by the TOC content of the river sediments. Even though the ecological risk assessment guidelines are chemical specific and do not establish causality where chemical mixtures occur, adverse ecological impacts are expected to occur mainly due to sediment contamination with p,p’–DDT and γ-HCH. Therefore, the government should set priority to control the use of DDTs and lindane in the Awash River Basin to prevent further contaminations.

## Supporting information

S1 Table Specific locations of the sampling sites. (PDF)

S2 Table OCPs concentration (ng g^-1^- dw) and TOC (%) of river sediments at specific sampling sites. (PDF)

S3 Table PBDEs and PCBs concentration (ng g^-1^- dw) of river sediments at specific sampling sites. (PDF)
