# Supplementary material for: Organochlorine pesticides, polybrominated diphenyl ethers and polychlorinated biphenyls in surficial sediments of the Awash River Basin, Ethiopia
Source: PLoS One. 2018 Oct 4;13(10):e0205026. doi: 10.1371/journal.pone.0205026 (PMC6171923; doi:10.1371/journal.pone.0205026)
Supplement: S1 Table — (PDF) [file pone.0205026.s001.pdf]

S1 Table: OCPs concentration (ng g^{-1}- dw) and TOC (%) of river sediments at specific sampling sites.

Site	α -HCH	β -HCH	γ -HCH	δ -HCH	p,p' -DDE	p,p' -DDD	p,p' -DDT	hep	hepe	aend	bend	endo	aldr	endr	enda	meth	TOC
1	1.329	8.911	1.540	0.243	1.741	0.790	7.258	28.425	27.451	0.273	n.d.	7.207	0.199	n.d.	1.501	1.348	0.005
2	1.016	2.413	1.022	0.129	0.190	0.572	4.327	26.094	27.516	0.186	0.661	1.905	0.116	0.500	1.797	1.136	0.039
3	1.046	3.633	1.040	0.384	0.234	0.718	1.048	28.389	23.577	0.234	n.d.	1.651	1.045	n.d.	n.d.	1.214	0.146
4	1.211	11.505	1.446	0.526	0.301	0.585	1.278	27.487	25.431	0.324	0.600	n.d.	0.759	n.d.	1.526	1.802	0.073
5	1.076	5.698	1.220	0.862	2.617	0.575	4.810	26.694	25.800	0.196	0.608	2.806	1.162	n.d.	1.515	2.220	0.041
6	0.99	7.04	1.03	0.16	1.14	1.78	3.15	24.53	30.53	0.67	0.60	2.35	1.18	1.33	1.13	2.50	0.310
7	1.548	4.557	n.d.	0.832	0.587	0.726	5.469	16.809	23.594	0.355	0.644	1.010	0.218	0.514	1.503	1.551	0.036
8	0.983	2.709	0.966	0.897	0.573	0.162	2.612	13.815	26.589	1.339	0.713	1.008	0.808	1.324	1.512	1.986	0.007
9	1.24	3.09	1.03	0.18	n.d.	1.05	2.49	24.38	27.59	0.57	0.60	1.50	0.86	1.35	0.76	1.26	0.009
10	0.88	0.83	0.84	n.d.	n.d.	0.81	1.96	22.38	15.27	0.65	0.62	1.63	0.57	1.33	0.53	2.93	n.d.
11	0.97	1.27	0.97	0.15	1.23	1.33	2.72	16.93	21.19	0.52	0.60	1.92	0.82	0.20	1.10	1.26	0.008
12	1.177	3.594	1.017	0.506	0.876	1.983	7.258	29.816	25.964	0.345	n.d.	1.000	0.967	0.508	n.d.	1.805	0.002
13	1.023	2.888	1.024	0.082	0.704	1.365	4.855	29.536	24.830	0.162	n.d.	3.601	1.128	n.d.	n.d.	1.216	n.d.
14	3.10	8.27	1.09	0.21	1.09	2.23	5.44	27.48	22.10	0.69	0.61	1.79	1.26	1.89	0.50	1.93	n.d.
15	1.23	2.61	0.92	0.26	1.00	1.85	3.84	23.98	36.29	n.d.	n.d.	1.64	0.59	1.35	0.56	1.23	0.035
16	1.83	6.37	1.09	0.18	0.68	1.62	4.94	29.50	15.39	0.61	0.60	1.90	1.04	1.34	0.50	1.26	n.d.
17	1.95	7.43	1.10	0.27	0.50	1.53	2.61	31.54	12.00	0.70	0.60	1.89	1.17	1.57	0.51	2.94	n.d.
18	1.98	7.07	1.04	0.16	0.58	1.01	3.22	31.59	36.29	0.55	0.60	1.99	0.57	1.33	1.22	1.29	0.002
19	1.36	3.21	1.01	0.22	0.52	2.08	2.79	24.00	36.29	0.56	n.d.	1.61	0.57	1.48	n.d.	1.39	0.009
20	1.85	6.77	1.07	0.20	0.49	0.91	3.16	27.72	9.80	n.d.	0.60	1.50	0.57	1.58	0.50	1.26	0.029
21	1.31	3.67	0.93	0.14	0.48	1.20	2.51	16.94	7.38	0.58	n.d.	1.66	2.31	1.34	0.77	1.79	0.030
22	1.34	3.73	1.01	0.18	0.52	0.75	2.45	33.31	29.09	0.60	0.60	1.93	0.86	1.53	0.81	n.d.	n.d.

S1 Table ….continued….

23	1.81	6.23	n.d.	n.d.	1.54	3.46	10.31	26.31	36.29	0.67	0.61	2.10	0.57	n.d.	0.87	1.27	0.126
24	1.92	7.20	n.d.	n.d.	0.61	1.12	6.23	26.92	25.34	0.57	n.d.	1.72	0.58	1.34	1.04	1.30	0.873
25	1.35	3.53	0.96	n.d.	0.38	0.75	2.77	15.50	17.10	n.d.	0.60	1.89	n.d.	1.34	0.78	1.26	0.020
26	1.75	5.61	0.29	0.27	1.49	29.31	108.87	19.87	36.29	n.d.	n.d.	2.08	0.58	n.d.	n.d.	n.d.	0.916
27	2.04	10.36	0.28	1.14	1.78	4.38	7.59	42.86	36.29	n.d.	n.d.	3.78	n.d.	n.d.	n.d.	n.d.	0.879
28	1.23	2.91	0.20	0.16	0.37	n.d.	2.45	25.24	22.81	n.d.	n.d.	n.d.	n.d.	1.35	0.73	1.26	0.769
29	1.23	12.24	1.49	n.d.	4.46	6.35	32.39	42.88	36.29	1.19	0.90	2.93	0.64	1.91	n.d.	1.30	1.893
30	1.40	3.87	1.01	n.d.	0.54	1.45	3.95	17.91	36.29	0.55	0.60	1.78	n.d.	1.38	0.50	1.31	0.912
31	1.30	3.35	0.97	0.13	0.87	1.45	8.57	13.36	11.12	0.54	0.60	1.67	n.d.	n.d.	0.71	1.26	0.918
32	0.86	0.71	0.84	n.d.	n.d.	0.76	1.60	n.d.	0.52	n.d.	n.d.	n.d.	n.d.	1.33	n.d.	n.d.	0.892
33	1.81	7.47	1.31	0.16	3.57	3.10	39.48	35.83	36.29	0.78	n.d.	2.42	n.d.	1.35	0.51	n.d.	1.593
34	1.59	5.57	0.23	0.16	1.81	1.84	13.89	28.47	28.12	0.61	n.d.	2.07	0.57	1.46	0.93	n.d.	1.705
35	1.06	1.92	0.25	n.d.	n.d.	1.14	4.78	8.45	3.06	0.53	0.60	1.58	0.57	1.34	0.62	1.26	0.900
36	1.66	7.04	1.10	n.d.	1.90	1.85	10.45	17.77	18.52	0.51	n.d.	1.91	n.d.	n.d.	0.81	n.d.	0.556
37	2.09	9.33	1.12	0.17	1.47	2.99	22.90	24.46	20.34	n.d.	0.61	1.83	n.d.	1.35	1.17	1.26	1.148
38	1.75	6.71	0.29	n.d.	1.24	2.39	14.70	17.52	19.43	0.55	0.60	2.38	n.d.	n.d.	1.19	n.d.	0.733
39	2.07	9.59	1.24	0.15	2.12	2.10	17.77	21.93	36.29	n.d.	0.61	n.d.	0.64	1.56	n.d.	n.d.	0.928
40	1.94	9.28	1.08	0.18	1.32	2.45	13.09	24.70	31.64	0.64	n.d.	1.84	0.57	1.59	1.28	1.30	0.310
41	1.24	3.01	1.12	n.d.	0.80	1.37	5.66	18.89	4.95	0.56	n.d.	n.d.	0.57	1.75	0.74	1.26	0.913
42	1.69	6.10	1.14	0.17	1.91	2.41	20.83	26.38	13.78	0.68	0.30	1.89	n.d.	1.85	0.96	0.63	0.702
43	1.37	4.63	1.07	0.15	1.47	1.83	17.23	18.34	19.22	0.61	n.d.	1.65	0.81	1.47	1.01	n.d.	1.243
44	1.96	8.30	1.07	0.16	1.70	1.98	10.96	15.31	23.73	0.70	n.d.	2.53	n.d.	1.59	1.10	n.d.	1.202
45	1.86	7.17	1.21	0.18	1.71	1.78	11.91	33.31	23.25	0.78	0.60	1.76	0.63	n.d.	1.13	1.26	0.588
46	2.57	11.97	1.36	n.d.	2.03	2.63	19.05	29.32	26.38	0.67	n.d.	1.99	0.58	1.55	1.45	1.27	1.303

Hep= heptachlor, **hepe=** heptachlor epoxide, **aend=**α-endosulfan, **bend=**β-endosulfan, **endo=** endosulfan-sulfate, **aldr=** aldrin, **endr=** endrin, **enda=** endrin-aldehyde, **meth** =methoxychlor
