# Supplementary material for: Organochlorine pesticides, polybrominated diphenyl ethers and polychlorinated biphenyls in surficial sediments of the Awash River Basin, Ethiopia
Source: PLoS One. 2018 Oct 4;13(10):e0205026. doi: 10.1371/journal.pone.0205026 (PMC6171923; doi:10.1371/journal.pone.0205026)
Supplement: S2 Table — (PDF) [file pone.0205026.s002.pdf]

S2 Table: PBDEs and PCBs concentration (ng g^{-1}- dw) of river sediments at specific sampling sites.

Site	Polybrominated diphenyl ethers							Polychlorinated biphenyls						
	**BDE-28**	**BDE-47**	**BDE-99**	**BDE-100**	**BDE-153**	**BDE-154**	**BDE-183**	**PCB-28**	**PCB-52**	**PCB-101**	**PCB-118**	**PCB-138**	**PCB-153**	**PCB-180**
1	1.539	1.840	0.893	0.793	0.774	1.000	n.d.	0.50	8.54	1.73	1.55	0.30	0.98	2.42
2	1.684	0.667	1.112	n.d.	0.704	1.175	1.000	0.86	1.58	2.02	0.30	0.68	0.96	0.85
3	1.351	0.755	1.237	0.682	0.716	1.274	0.999	0.40	3.14	0.30	0.30	0.35	0.98	4.51
4	1.617	0.758	0.887	1.223	0.810	n.d.	1.204	1.40	6.54	2.51	0.72	0.35	1.69	2.55
5	2.217	0.668	0.667	0.776	0.891	1.439	1.014	0.42	2.37	0.31	0.30	0.54	0.46	0.90
6	1.00	0.77	0.67	0.67	0.73	1.00	n.d.	n.d.	2.08	n.d.	0.45	n.d.	0.69	1.51
7	1.775	4.113	1.760	0.824	0.851	1.056	0.983	0.41	0.36	0.46	0.31	0.35	4.80	3.45
8	1.259	6.508	1.540	0.833	0.667	n.d.	1.888	1.46	4.70	2.78	0.81	0.85	1.78	6.45
9	1.00	0.67	1.18	0.67	0.72	1.00	2.68	1.80	1.79	1.88	0.50	0.35	0.45	1.26
10	n.d.	3.13	1.91	0.67	0.70	1.00	2.88	n.d.	1.00	n.d.	0.50	0.35	n.d.	1.04
11	1.00	0.67	0.75	0.68	0.73	1.00	1.36	n.d.	2.77	n.d.	0.48	n.d.	0.44	1.02
12	1.270	1.502	1.133	0.788	0.692	n.d.	0.998	4.84	5.63	0.30	0.30	0.35	1.06	3.42
13	1.521	1.404	1.506	0.692	0.742	1.129	0.980	1.96	4.64	1.79	0.28	0.67	0.77	1.32
14	1.00	0.95	1.31	0.67	0.75	1.00	2.35	n.d.	3.34	3.04	0.50	0.34	0.62	1.79
15	n.d.	0.68	0.67	0.68	0.68	1.02	n.d.	2.24	2.49	1.02	n.d.	n.d.	0.46	1.86
16	1.00	0.67	0.90	0.68	0.79	1.00	2.10	2.90	2.22	2.39	0.51	0.35	0.59	1.76
17	1.01	0.67	0.91	0.67	0.74	1.02	1.79	3.70	2.73	2.83	0.48	2.04	0.69	n.d.
18	n.d.	0.68	0.67	0.67	0.77	1.00	n.d.	2.58	3.37	2.59	0.56	n.d.	0.43	1.27
19	n.d.	0.747	0.71	0.68	0.68	1.02	n.d.	2.36	4.46	1.12	0.50	n.d.	0.35	1.35
20	1.00	0.79	0.82	0.67	0.72	1.02	2.06	n.d.	2.30	2.48	0.61	0.84	0.45	1.25
21	1.04	0.77	0.80	0.67	0.73	1.00	2.87	1.54	1.76	0.69	0.49	1.37	0.48	1.36
22	1.00	0.68	0.80	0.68	0.73	1.01	n.d.	2.27	2.23	2.32	0.50	n.d.	0.45	1.09

S2 Table….continued….

23	1.01	0.68	0.68	0.75	0.67	1.00	2.66	2.17	6.69	2.04	0.50	0.35	0.53	1.36
24	1.01	0.65	0.69	0.66	1.21	1.00	2.16	2.30	10.49	2.75	0.50	0.35	0.39	1.28
25	1.00	0.67	0.67	0.67	0.75	n.d.	3.74	1.33	7.08	1.75	0.50	0.35	n.d.	1.20
26	2.01	0.66	2.59	0.68	0.65	1.04	n.d.	2.84	16.79	1.17	n.d.	0.35	n.d.	2.05
27	n.d.	10.61	5.91	0.66	0.76	1.01	n.d.	n.d.	18.91	2.50	n.d.	0.35	0.46	4.34
28	1.00	0.68	0.68	0.68	0.74	1.00	3.62	1.59	7.33	1.73	0.48	0.33	0.45	1.26
29	1.00	0.66	0.61	0.64	0.84	1.00	2.46	3.91	5.66	2.68	0.51	n.d.	n.d.	2.74
30	n.d.	0.67	0.67	0.68	0.99	1.01	2.92	1.64	1.41	1.82	0.50	0.35	n.d.	1.31
31	n.d.	0.66	0.67	0.66	0.89	1.00	4.12	1.08	n.d.	1.47	0.50	0.35	0.35	1.15
32	1.00	0.67	0.67	0.67	0.67	1.00	1.38	n.d.	n.d.	n.d.	0.50	0.35	n.d.	n.d.
33	1.19	0.67	0.66	0.68	0.66	n.d.	2.08	2.65	3.42	n.d.	0.49	n.d.	n.d.	1.22
34	1.11	0.67	0.81	0.67	0.76	1.00	4.96	1.79	1.45	1.69	0.49	0.35	0.37	1.11
35	1.00	0.67	0.70	0.67	0.67	1.00	2.90	n.d.	n.d.	0.72	0.50	0.35	0.38	1.02
36	n.d.	0.61	0.63	0.55	0.68	n.d.	1.23	1.23	1.28	1.87	0.50	0.35	0.41	1.28
37	1.00	0.68	0.66	0.67	0.78	1.00	1.69	1.49	1.73	3.07	0.51	0.35	0.64	1.54
38	1.00	0.69	0.67	0.67	0.76	1.00	2.21	2.44	1.48	2.09	0.50	0.35	n.d.	n.d.
39	1.07	0.66	0.67	0.66	0.76	1.00	1.66	1.82	1.14	1.30	0.51	n.d.	n.d.	1.30
40	0.91	0.66	0.68	0.67	0.82	n.d.	n.d.	2.29	2.12	3.75	0.50	0.35	0.77	1.94
41	1.09	0.67	0.67	0.68	0.70	1.00	2.17	1.34	5.29	1.40	0.50	0.61	0.37	1.08
42	1.16	0.67	0.76	0.67	0.80	1.00	2.26	2.76	2.25	1.99	0.49	0.35	0.48	1.28
43	n.d.	0.67	0.67	0.70	0.67	1.00	3.04	1.86	1.58	1.59	0.50	0.35	0.39	1.02
44	1.00	0.67	0.67	0.67	0.76	1.04	2.05	1.77	1.20	1.91	0.50	n.d.	0.45	1.47
45	1.15	0.66	0.91	0.74	0.80	n.d.	1.39	3.13	3.22	2.87	0.50	0.35	0.53	1.43
46	1.00	0.66	0.67	0.67	0.76	1.00	1.41	2.36	2.29	2.93	0.50	0.35	0.59	1.85
