# Supplementary material for: Organochlorine pesticides, polybrominated diphenyl ethers and polychlorinated biphenyls in surficial sediments of the Awash River Basin, Ethiopia
Source: PLoS One. 2018 Oct 4;13(10):e0205026. doi: 10.1371/journal.pone.0205026 (PMC6171923; doi:10.1371/journal.pone.0205026)
Supplement: S3 Table — (PDF) [file pone.0205026.s003.pdf]

S3 Table : Specific locations of the sampling sites.

Sites	Altitude	Latitude	Longitude	Sites	Altitude	Latitude	Longitude
1	933.5	8.838056	40.01386	24	1814	8.624778	38.748
2	935.2	8.841167	40.01119	25	1806	8.624972	38.75136
3	950.5	8.862083	40.01772	26	2058	8.841694	38.77911
4	958.4	8.803083	39.89133	27	2061	8.876389	38.7845
5	987.6	8.758944	39.88472	28	2163	8.943389	38.77222
6	997.3	8.757528	39.882	29	2384	9.032472	38.70117
7	1154	8.645556	39.75086	30	2384	9.028694	38.77958
8	1148	8.650361	39.75344	31	2017	8.706333	38.59508
9	1242	9.038806	39.56486	32	1995	8.704556	38.60458
10	1248	9.041361	39.562	33	2067	8.84675	38.41008
11	1394	8.383556	39.37706	34	2063	8.849667	38.4075
12	1341	8.40375	39.3905	35	2064	8.860917	38.35703
13	1552	8.445056	39.25956	36	2149	8.605556	38.208
14	1546	8.468167	39.23372	37	2148	8.684	38.20572
15	1588	8.465194	39.15358	38	2181	8.789536	38.13753
16	1592	8.405472	39.02097	39	2149	8.768556	38.14928
17	1605	8.430111	39.02214	40	2130	9.017639	38.26486
18	1748	8.597472	39.11111	41	2124	9.022694	38.35125
19	1712	8.572944	39.10853	42	2126	9.019528	38.35086
20	1760	8.612833	39.11444	43	2225	9.019806	38.50181
21	1789	8.641306	39.11175	44	2229	9.023472	38.50678
22	1842	8.69425	39.08275	45	2391	9.073667	38.508
23	1804	8.618861	38.75072	46	2382	9.073	38.51042
